# Lipid Profiling of Four Guava Cultivars: A Multi-Dimensional Spatial Analysis

**DOI:** 10.3390/foods14132330

**Published:** 2025-06-30

**Authors:** Qun Zhang, Xueren Cao, Yujun Ding, Chen Ma, Qiong Fan, Jia Song, Yu Rong, Di Chen, Wenjiang Dong, Xiaopeng Wu, Zhi Xu, Daizhu Lyu

**Affiliations:** 1 Analysis and Test Center, Chinese Academy of Tropical Agricultural Sciences, Key Laboratory of Quality and Safety Control for Subtropical Fruit and Vegetable, Ministry of Agriculture and Rural Affairs, Hainan Provincial Key Laboratory of Quality and Safety for Tropical Fruits and Vegetables, Key Laboratory of Nutritional Quality and Health Benefits of Tropical Agricultural Products of Haikou City, Haikou 571101, China; zhangqun123@zju.edu.cn (Q.Z.); dyjyj0822@163.com (Y.D.); mc19860112@163.com (C.M.); joanhee@126.com (Q.F.); jia668837@163.com (J.S.); amanda941130@163.com (Y.R.); chendi_1008@yeah.net (D.C.); dygxzx@126.com (X.W.); 2 Ministry of Agriculture, Environment and Plant Protection Institute, Chinese Academy of Tropical Agricultural Sciences, Key Laboratory of Integrated Pest Management on Tropical Crops, Haikou 571101, China; caoxueren1984@163.com; 3 Spice and Beverage Research Institute, Chinese Academy of Tropical Agricultural Sciences, Haikou 571101, China; dongwenjiang.123@163.com

**Keywords:** *Psidium guajava* L., UPLC-MS/MS, lipids, metabolic pathways

## Abstract

This study aimed to reveal the lipid composition and distribution and characterize the lipid metabolism profile in the three distinct parts of four guava varieties with varying textures and colors using liquid chromatography–electrospray ionization–tandem mass spectrometry. The four varieties, collected from a guava cultivation base in Danzhou City, Hainan Province, were “Zhenzhu” (white-fleshed hard-crispy guava, YBSL), “Bendi” (white-fleshed soft-waxy guava, RBSL), “Xiguahong” (red-fleshed hard-crispy guava, YHSL), and “Hongxin” (red-fleshed soft-waxy guava, RHSL). A total of 8242 lipids were detected, which were classified into four categories and 20 subcategories. Glycerolipids and glycerophospholipids are the most abundant types of lipids in guava. The lipid composition showed significant differences between hard-crispy and soft-waxy guavas. The red-fleshed guava varieties had 98, 57, and 96 differential lipid metabolites, whereas white-fleshed varieties had 68, 108, and 41 lipid metabolites in the epicarp, mesocarp, and endocarp, respectively. Moreover, comparative analysis of hard-crispy versus soft-waxy guavas with different colors revealed common differential lipids in the epicarp (29), mesocarp (21), and endocarp (18). The common differential lipids, including phosphatidylcholine (PC) (16:0/18:1), PC (18:1/18:1), and phosphatidylethanolamine (PE) (18:1/18:2), were found to be upregulated across all fruit parts, with greater abundance in soft-waxy guavas. They were mainly enriched in metabolic pathways associated with glycerophosphocholine and glycerophosphoethanolamine. These differential lipids may serve as potential biomarkers for evaluating guava quality. This study unveiled the lipid distribution and metabolic variations among different guava varieties. It also established a scientific foundation for improving guava varieties and implementing quality control measures.

## 1. Introduction

Guava (*Psidium guajava* L.) is a tropical specialty fruit belonging to the genus *Psidium* in the Myrtaceae family. It contains significant amounts of bioactive compounds, including polyphenols, flavonoids, tannins, terpenes, saponins, carotenoids, and polysaccharides [1,2,3,4]. It has gained increasing attention worldwide, with increasing demand due to its antioxidant, anti-inflammatory, immune-modulatory, antimicrobial, antidiabetic, and cancer-fighting properties [5,6,7,8]. Recent studies have focused on the nutritional composition, including acids, amino acids, minerals, pigments, proteins, polyphenols, sugars, vitamin C, and flavor compounds, of guava [6,9,10,11]. The primary organic acids present in guava fruits include ascorbic acid, which is measured at 196.5 mg per 100 g, and titratable acidity, quantified as 0.64% citric acid [9]. Both ascorbic acid and titratable acidity levels decrease in the immature and intermediate stages of guava fruit maturation [9]. Guava varieties can be classified into two main categories based on taste: hard-crispy and soft-waxy. They can also be divided into red-flesh and white-flesh types based on the color of the flesh. Recent studies have demonstrated that guava varieties with different tastes exhibit varying concentrations of ascorbic acid, overall phenolic levels, antioxidative capabilities, and fruit aromas [12,13]. However, no comprehensive identification or comparative analysis of lipid profiles in different guava varieties has been conducted yet.

Lipids play a crucial role in plant evolution, ripening, and fruit quality. Also, they are essential for maintaining plant cell structure, facilitating energy storage, and enabling signal transmission. Recently, a decrease in triacylglycerol (TAG) levels has been reported in avocado seeds and mesocarps. This suggests a primary role of TAGs in providing acetogenin during fruit growth, thereby influencing the nutritional and flavor changes in the fruit [14]. Furthermore, reducing phospholipid breakdown, increasing phosphatidic acid accumulation, and minimizing lipid peroxidation in cell membranes are significant factors mitigating the internal browning of pineapple fruit, thus preserving its quality [15]. Further, lysophosphatidylethanolamine 18:1, which is inhibited by fatty acid desaturase-2, exhibits a positive correlation with fruit firmness [16]. However, the success of these research endeavors hinges on the use of advanced, high-efficiency analytical methods. Accurate identification and quantification of lipid components within complex matrices can be effectively achieved using ultra-high-performance liquid chromatography–tandem mass spectrometry (UPLC-MS/MS) based on the multiple reaction monitoring (MRM) model [17,18,19,20]. Recent years have witnessed the successful application of this technology in apple [21], banana [22], pineapple [15], grapes [23], wheat [24], and tobacco leaves [19]. However, the composition and distribution of lipids in guavas remain unclear, particularly in different varieties and parts of the fruit.

In this study, guava fruits were segregated into three distinct morphological components, epicarp, mesocarp, and endocarp, reflecting their berry characteristics. Based on this segmentation, the study aimed to achieve the following: (1) characterize the lipid metabolism profiles in these three distinct parts across four guava varieties using UPLC-MS/MS, (2) reveal the lipid composition and distribution among different guava varieties, and (3) identify the core differentially expressed lipid molecules and the associated enriched pathways. The results of this study may provide a scientific basis for improving guava cultivation practices and enhancing quality control during processing.

## 2. Materials and Methods

### 2.1. Instruments, Reagents, Materials, and Samples

This study used a Triple Quad™ 6500 6500 ultra-high-performance liquid chromatography (HPLC)–tandem mass spectrometer (AB SCIEX Corporation, Framingham, MA, USA), a Kinetex C18 HPLC column (2.6 µm, 2.1 mm × 100 mm, Phenomenex Inc., Tianjin, China), an AL204 analytical balance with the sensitivity of one ten-thousandth of a gram (Mettler-Toledo Instruments (Shanghai) Co., Ltd., Shanghai, China), a CR22N floor-standing high-speed refrigerated centrifuge (Hitachi Ltd., Tokyo, Kanto Area, Japan), an ultrapure water system (MILLIPORE Corporation, Burlington, MA, USA), a T25 basic high-speed homogenizer (Guangzhou Yike Experimental Technology Co., Ltd., Guangzhou, Guangdong, China), a digital caliper (Hangzhou Delixi Group Co., Ltd., Hangzhou, Zhejiang, China), and a TA-XT Plus texture analyzer (Stable Micro Systems Ltd., Godalming, Surrey, UK).

Acetonitrile and methanol of mass spectrometry quality were obtained from Fisher Scientific (Waltham, MA, USA).

Four different varieties of *Psidium guajava* L. were collected from a guava cultivation base in Danzhou City, Hainan Province: “Zhenzhu” (white-fleshed hard-crispy guava, YBSL), “Bendi” (white-fleshed soft-waxy guava, RBSL), “Xiguahong” (red-fleshed hard-crispy guava, YHSL), and “Hongxin” (red-fleshed soft-waxy guava, RHSL). All the fruits were ripe and of uniform size, with no visual defects.

### 2.2. Sample Preparation and Analysis of Properties

#### 2.2.1. Sample Preparation

Ten guava specimens were selected from every variety after cleaning and desiccating the fruits. The epicarp, mesocarp, and endocarp of the fruits were separated and then homogenized individually. The seeds were intentionally retained as part of the endocarp fraction during sample preparation, as they represent an integral component of guava biomass in industrial processing [25]. Approximately 10 g of homogenized part samples were delicately pulverized in a nitrogen solution and immediately stored at −80 °C until analysis.

#### 2.2.2. Assessment of Physical Properties

The fresh weight of each guava was measured with a digital weighing device. A vernier caliper was used to gauge the length and width of fruits. The fruit shape index was calculated as the ratio of fruit height and width. The firmness of each guava was determined with a texture analyzer by performing a puncture examination on the equatorial side of fruits. A cylindrical probe with a diameter of 2 mm was used, operating at a penetration rate of 2 mm/s to a depth of 10 mm. The results were represented as the mean ± standard error.

#### 2.2.3. Evaluation of Sensory Properties

The sensory properties of guava fruits, including visual inspection, color, and texture, were evaluated. The visual inspection was conducted by trained assessors following the methodologies outlined by Omayio et al. [26] and Khan et al. [27]. The peel color was classified as either green or yellowish-green, whereas the pulp color was categorized as white or red in accordance with the cultivar standards. The texture classification involved categorizing fruits as “hard-crispy” or “soft-waxy” based on tactile evaluation.

### 2.3. Sample Extraction

The sample was extracted as described by Su et al. [22] with some modifications. Initially, 150 mg of the sample was transferred into an Eppendorf (EP) tube containing 600 μL of water. The mixture was vortexed for 30 s and then ultrasonicated in ice water for 15 min. A 1440 μL of extraction solvent (methyl tert-butyl ether/methanol = 5:1) was added to the mixture, and the mixture was vortexed for 30 s, followed by ultrasonication in ice water for 5 min. The final step involved transferring 1520 μL of the supernatant into a 2 mL centrifuge tube. The sample was dried with nitrogen purging and reconstituted by adding 800 μL of solution (dichloromethane/methanol = 1:1), vortexed for 30 s, and then ultrasonically treated with water for 10 min. Subsequently, the sample was centrifuged at 4 °C and 12,000 rpm for 15 min, and 600 μL of supernatant was placed in a vial. Finally, 20 μL of each sample’s injection solution was used to generate a quality control (QC) sample. Six replicates were prepared for each treatment.

### 2.4. UPLC-MS/MS Analysis

#### 2.4.1. Chromatographic Method

For the positive ion source, phase A comprised a balanced mixture of water, methanol, and acetonitrile (*v*/*v*/*v*) with 7 mM ammonium acetate, whereas phase B contained isopropanol at the same concentration of ammonium acetate. Details of gradient elution conditions of the mobile phase are provided in Table S1. The injection volume was 1 μL, and the column temperature was 45 °C. In contrast, for the negative ion source, phase A contained a mixture of isopropanol and acetonitrile in a ratio of 7:93 (*v*/*v*) with 5 mM ammonium acetate, while phase B contained a mixture of water and acetonitrile in a ratio of 1:1 (*v*/*v*) with 2 mM ammonium acetate. Details of gradient elution conditions of the mobile phase are provided in Table S2. The injection volume was 1 μL, and the column temperature was 35 °C.

#### 2.4.2. Mass Spectrometry Method

For the positive ion source in mass spectrometry, the parameters were set as follows: curtain gas at 40 psi, nebulizer gas at 55 psi, auxiliary heating gas at 55 psi, ion spray voltage of 5200 V, and ion source temperature at 350 °C. In contrast, the negative ion source had curtain gas at 40 psi, nebulizer gas at 50 psi, auxiliary heating gas at 50 psi, and ion spray voltage at −4500 V with the ion source temperature at 350 °C. For improved measurement of lipids, our lab group created a reference collection using MS/MS data [22], encompassing names of lipids, measured mass-to-charge (*m*/*z*) ratios, and their retention intervals. We made it easier to identify lipids by analyzing their retention duration (RT) and *m*/*z* ratio. We employed SCIEX OS version 1.4.0.18067 and MultiQuant software version 3.02 for peak detection and filtering.

### 2.5. Statistical Analysis

A one-way analysis of variance and independent sample Tukey’s tests were performed using SAS 9.0 to determine the significance of differences in sensory properties between different varieties of guava at a 95% confidence level (*p* ≤ 0.05). The lipidomic data were compared among different groups using the MetaboAnalyst 6.0 platform. The group clustering was performed employing multivariate statistical methods, including principal component analysis (PCA). Univariate statistical analysis was used, particularly *t* tests for *p* values, to reveal the variations in fold changes. The lipid molecules were chosen by considering |FC| ≥ 2 and *p* < 0.05. Significant lipid metabolites were then identified using variable importance in projection (VIP) analysis, with a threshold of VIP ≥ 1.0. For further pathway enrichment analysis, a *p* value of less than 0.05 was considered indicative of significant enrichment.

## 3. Results

### 3.1. Morphological and Physical Characteristics of Guava Varieties

**Figure 1:**
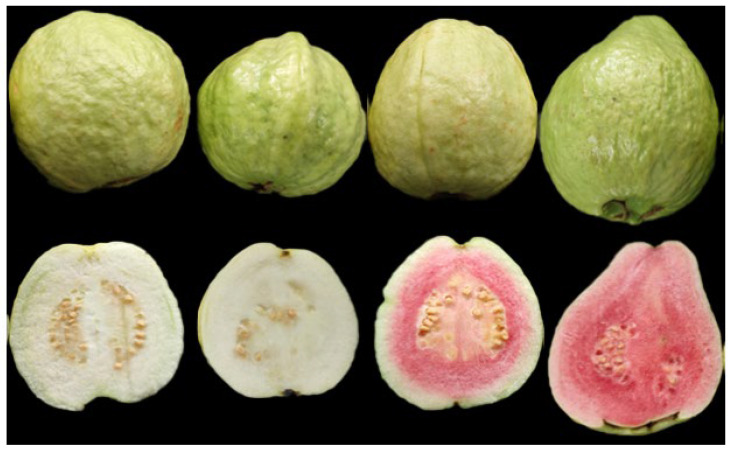
Morphological characteristics of four varieties of guava fruit. Note: YBSL: white-fleshed hard-crisp guava; RBSL: white-fleshed soft-waxy guava; YHSL: red-fleshed hard-crisp guava; RHSL: red-fleshed soft-waxy guava.

The morphological and physical characteristics of the guavas, particularly the fruit shape index, fruit weight, firmness, and flesh color, were obviously different (Figure 1 and Table 1). The fruit horizontal diameter ranged from 6.26 cm (YHSL variety) to 7.28 cm (RHSL variety) among the four selected varieties. The variation in the fruit vertical diameter ranged from 6.22 cm (RBSL variety) to 8.10 cm (RHSL variety). In this study, the fruit shapes of YHSL (1.02) and YBSL (1.01) were round, matching the evaluated fruit shape index. In contrast, RBSL were oval-shaped, whereas RHSL were pear-shaped. These findings were consistent with those of Omayio et al. [26] and Khan et al. [27], who examined the physiochemical properties of various guava cultivars. The fruit weight ranged from 132.92 to 179.56 g, with the RHSL having the highest fruit weight and the RBSL having the lowest fruit weight. Moreover, the fruit firmness varied across the guava varieties in the following order: YHSL > YBSL > RHSL > RBSL. However, despite having similar fruit textures, red-fleshed guava exhibited significantly greater firmness than that of white-fleshed guava. These results revealed a broad variation in morphological and physical characteristics among various guava varieties.

### 3.2. Determination of Lipid in Different Fruit Parts of Guava Varieties

**Figure 2:**
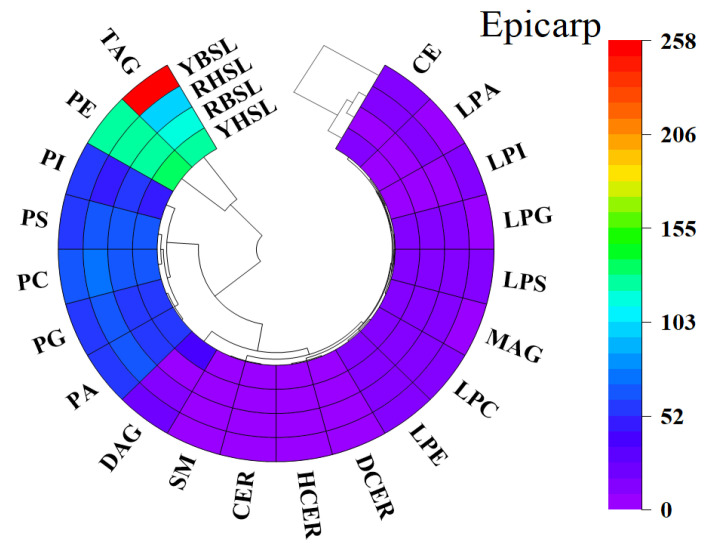

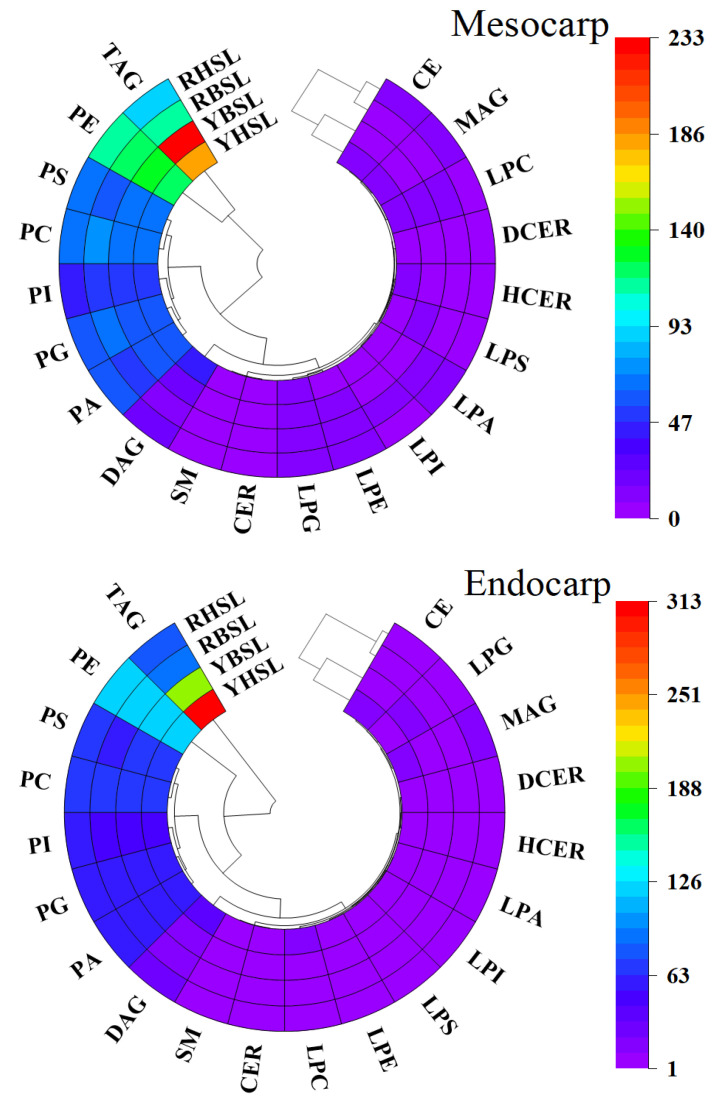
Clustering heat map of the detected numbers of lipid subclasses in different parts of guava. Note: PE, phosphatidylethanolamine; PS, phosphatidylserine; PC, phosphatidylcholine; PG, phosphatidylglycerol; PA, phosphatidic acid; PI, phosphatidylinositol; LPC, lysophosphatidylcholine; LPE, lysophosphatidylethanolamine; LPG, lysophosphatidylglycerol; LPS, lysophosphatidylserine; LPA, lysophosphatidic acid; LPI, lysophosphatidylinositol; TAG, triacylglycerol; DAG, diacylglycerol; MAG, monoacylglycerol; HCER, hexosylceramide; DCER, dihexosylceramide; CER, ceramide; SM, sphingomyelin; CE, cholesterol ester.

Targeted metabolic profiling was performed using UPLC-MS/MS to investigate the lipid metabolites across different parts of the four guava varieties. A total of 8242 lipid metabolites were detected and categorized into four different classes: glycerophospholipids (GPs), glycerolipids (GLs), sphingolipids (SPs), and sterols (STs) (Figure 2 and Table S3). Also, these lipids were divided into 20 distinct subcategories. It indicated that the lipid profile in the epicarp of YBSL guava differed significantly from those of the other three varieties. In contrast, the mesocarp and endocarp of the four guavas could be categorized into two main groups: hard-crispy and soft-waxy. The same lipid classification was observed across various parts of the four guava varieties. Among these, GPs were the predominant lipids in guava, with a range starting from 53.99%. These findings aligned with previous studies demonstrating that GPs were the most abundant lipids in bananas [22], grapes [23], and fresh walnut kernels [28]. Also, across all fruit parts, soft-waxy guava had a higher proportion of GPs compared with hard-crispy guava. This result indicated that a higher proportion of GPs was correlated with lower fruit firmness in guava fruits. GPs are the major components of the cell wall, and the structural composition of the cell wall is closely related to the textural properties of fruits [29,30]. The high mobility of GPs within the cell wall can increase membrane flexibility, thus potentially decreasing the overall firmness of fruits [31]. Therefore, GPs in guava play a significant role in determining texture.

Further analysis revealed significant differences in the numbers of triacylglycerols (TAGs) detected across various parts of the four guava varieties (Figure 2 and Table S3). In YHSL guava, a higher number of TAGs were detected in the endocarp than in other parts of the fruit. However, in the YBSL, RBSL, and RHSL guavas, the number of tags detected in the endocarp was lower than in other parts of the fruit. However, it is important to note that seeds were included in the endocarp fraction of our analysis, which may influence the overall lipid profile due to their known high lipid content, particularly TAGs [25,32]. Although this approach reflects realistic biomass utilization scenarios, future studies could benefit from employing component-specific extraction methods to further distinguish seed-derived lipid contributions from those of other fruit parts. Similarly, a higher proportion of TAGs were detected in the endosperm of Costa Rican *Acrocomia aculeata* fruits than in the mesocarp [33]. Moreover, a significant difference was noted between mesocarp and seed in the distribution of TAGs in olive fruit (*Olea europaea* L.) [34]. These findings underscored the importance of analyzing lipid composition across various fruit parts.

### 3.3. PCA for Each Group and QC Samples

**Figure 3:**
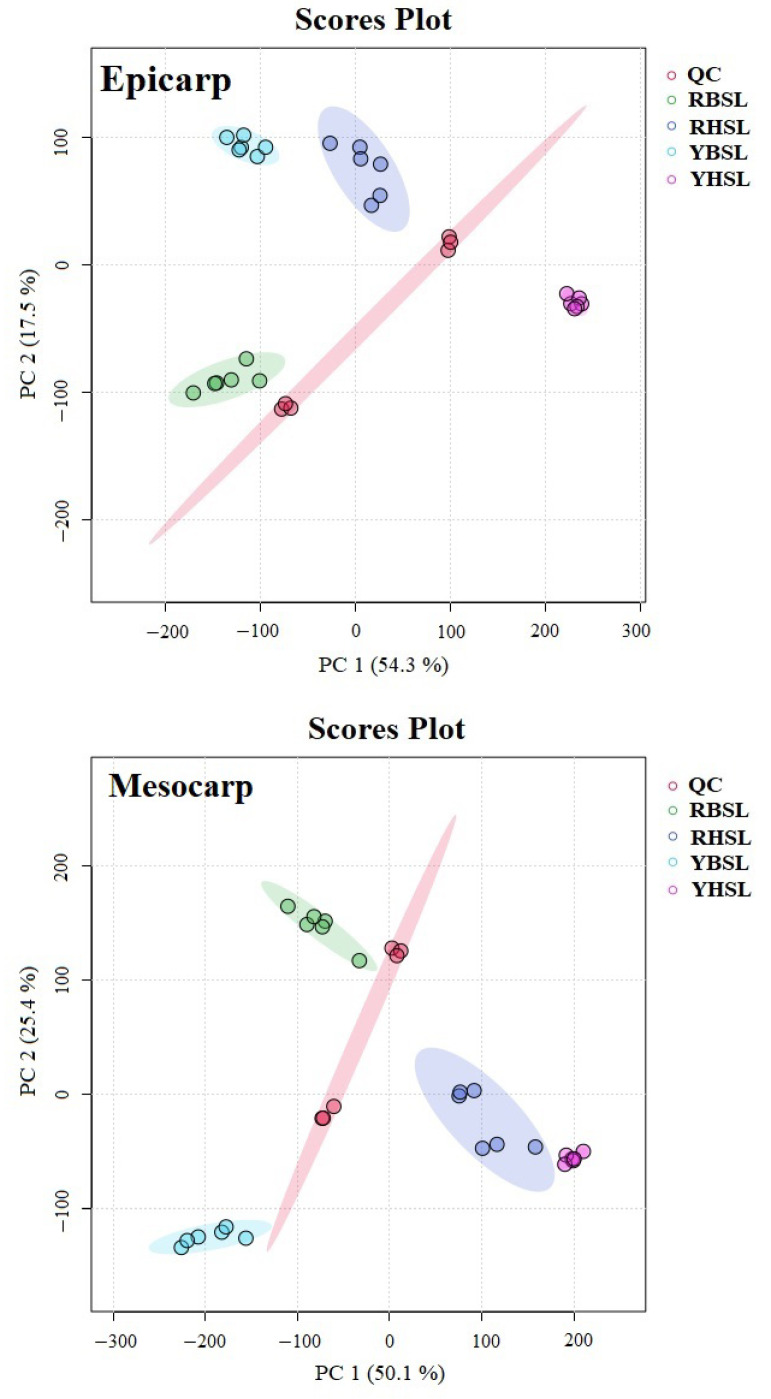

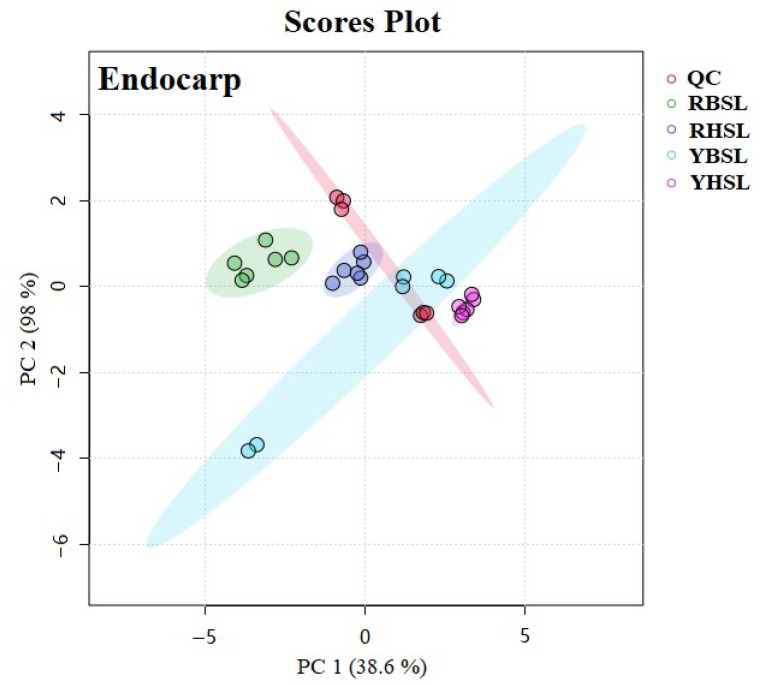
PCA score map of lipid distribution in different parts of guava.

The peak intensities were transformed into unit percentages and then applied for multivariate statistical analysis to evaluate the significant differences in lipid content in the four guava cultivars. PCA was used to assess both within-group and between-group variations, as described previously [15,22,35]. A tendency for separation was noted in the lipid compositions of the epicarp, mesocarp, and endocarp of the four guava cultivars (Figure 3). Particularly, in the mesocarp, the first principal component (PC1) differentiated the white-fleshed guava cultivar from the red-fleshed guava cultivar. The QC samples demonstrated consistent lipid patterns throughout this study, confirming methodological reliability.

### 3.4. Differential Lipid Analysis of the Epicarp, Mesocarp, and Endocarp of Hard-Crispy and Soft-Waxy Guavas with Different Flesh Colors

**Figure 4:**
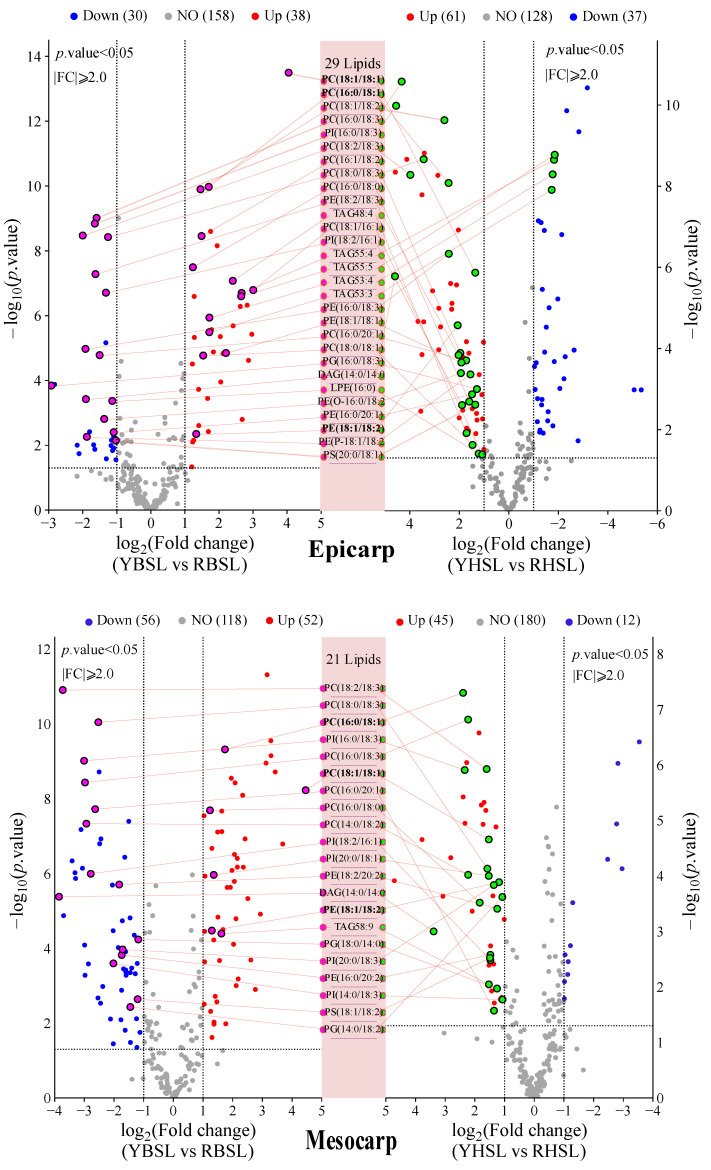

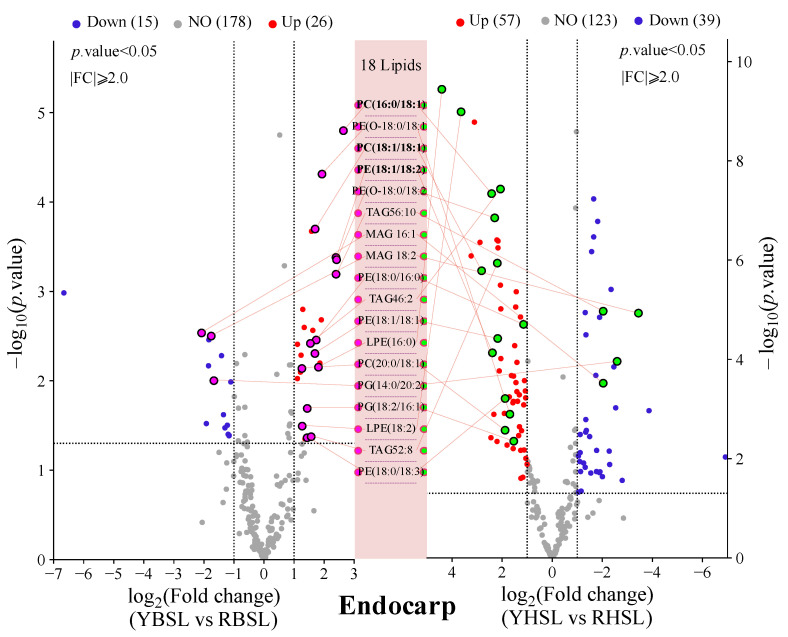
Different lipid volcanic maps of different parts of comparison groups (YBSL vs. RBSL and YHSL vs. RHSL). Note: purple dots, the common differential lipids upregulated and downregulain YBSL vs. RBSL; green dots, the common differential lipids upregulated and downregulain YHSL vs. RHSL.

The lipid volcanic maps for various guava parts across comparison groups (YBSL vs. RBSL and YHSL vs. RHSL) are depicted in Figure 4. Comparing YHSL with RHSL identified 98 differential lipids, with 61 upregulated and 37 downregulated. A similar trend was observed in the endocarp as in the epicarp. However, an inverse situation was noted in the mesocarp. The comparison of YBSL vs. RBSL revealed 68 differential lipids, with 38 upregulated and 30 downregulated. The findings suggested that the lipid changes in the endocarp and epicarp of red-fleshed guava, which were attributed to the variations in fruit firmness, were notably greater than those in white-fleshed guava. In contrast, the variations in lipid composition were more distinct in the mesocarp of white-fleshed guavas with different firmness levels compared with red-fleshed guavas. Furthermore, the number of downregulated lipids was higher than that of upregulated lipids (up/down ratio 0.93) in the mesocarp of YBSL versus (vs.) RBSL. However, in other comparison groups, the number of upregulated lipids exceeded that of downregulated lipids, with the up/down ratio ranging from 1.1 to 3.7. All these results indicated significant differences in lipid profiles among different fruit parts when comparing hard-crispy with soft-waxy guavas of varying flesh colors.

A comparative analysis of YHSL vs. RHSL and YBSL vs. RBSL was conducted to further elucidate the common differences in lipid composition between the two groups of hard-crisp and soft-waxy guavas with varying flesh colors (Figure 4 and Table S4). It revealed 29 common differential lipids in the epicarp, decreasing to 21 in mesocarp and further reducing to 18 in endocarp analyses. Moreover, it demonstrated that the major upregulated lipids were GPs. The epicarp showed 10 upregulated lipids: nine GPs and one GL. The mesocarp exhibited six upregulated lipids: five GPs and one GL. The endocarp displayed 14 upregulated lipids: 12 GPs and two GLs. Additionally, downregulation of three lipids was observed in the endocarp, comprising two GLs [monoacylglycerol (MAG) 16:1 and MAG 18:2] and one GP [phosphatidylglycerol (PG) (14:0/20:2)]. GPs were significantly more abundant than GLs across all parts, thus underscoring the crucial role of GPs in the lipid composition of guava.

**Figure 5:**
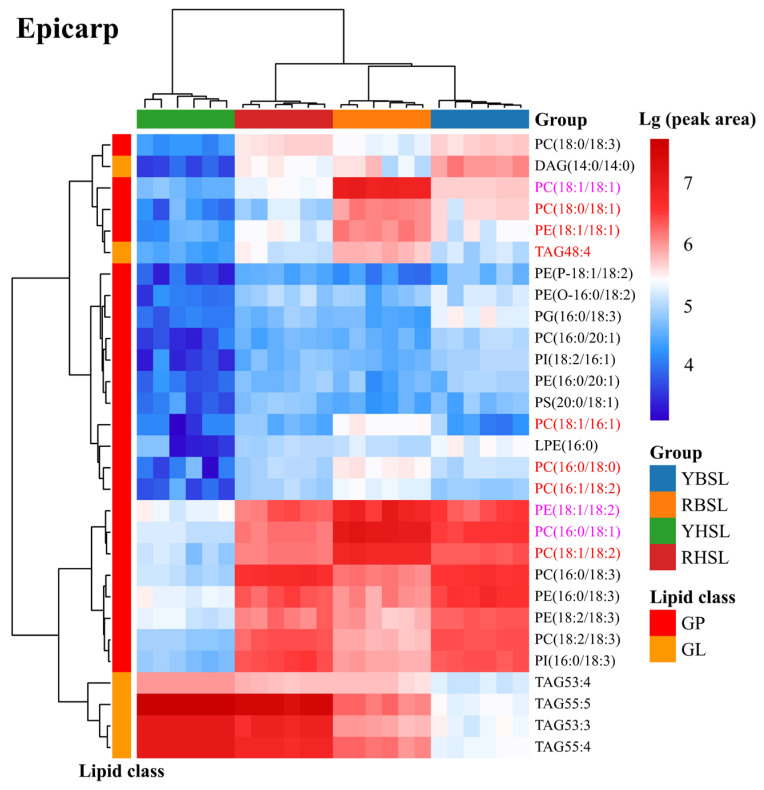

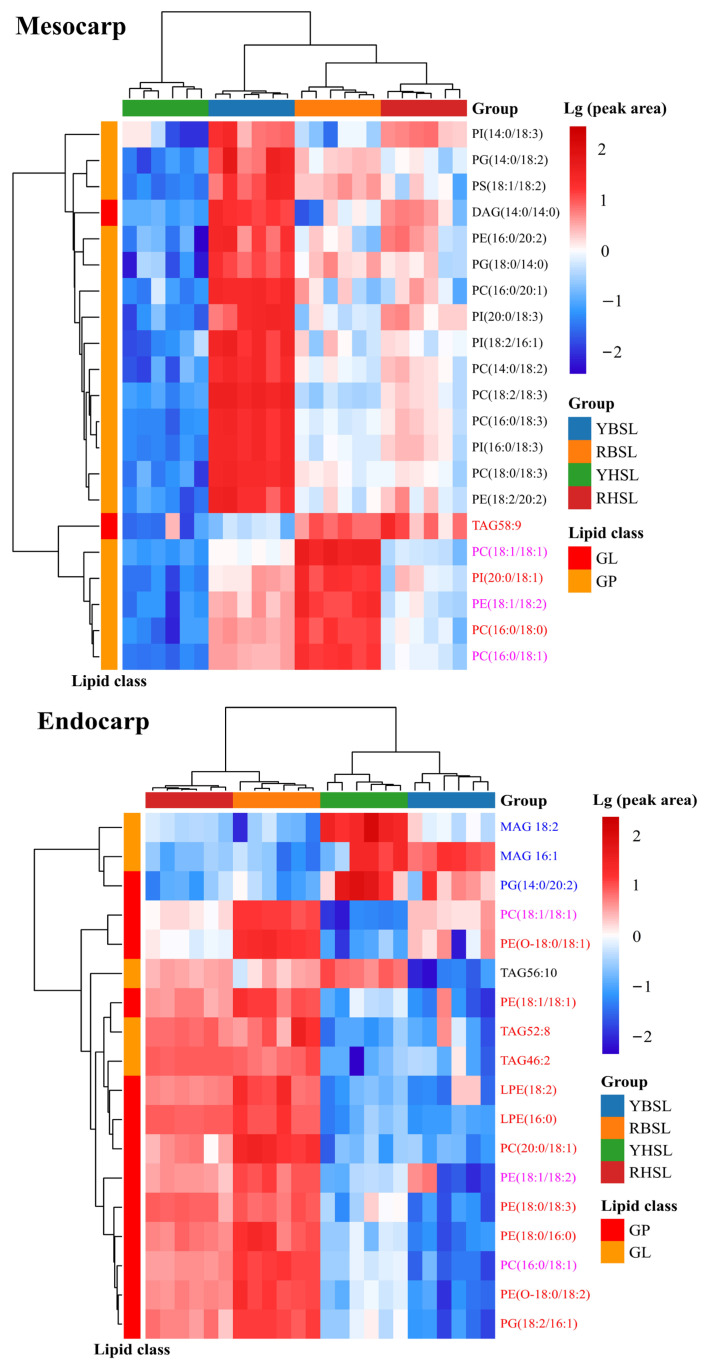
The cluster heat maps of different parts of comparison groups (YBSL vs. RBSL and YHSL vs. RHSL). Note: The lipids with names highlighted in red font are commonly upregulated, those in purple font are commonly upregulated and present in all guava parts, and those in blue font are commonly downregulated.

The logarithmic transformation and normalization of lipid peak areas were conducted to further clarify the differences in lipids. Subsequently, a clustering heatmap was generated to categorize all samples into four distinct groups based on lipid composition (Figure 5). Significant differences were observed among the various guava varieties. In particular, the lipid profiles of the YHSL variety differed notably from those of the other three varieties in the epicarp and mesocarp. This finding underscored the uniqueness of the lipid composition in the epicarp and mesocarp of the YHSL variety. While analyzing the lipid composition in the endocarp, the samples from the YBSL and YHSL varieties were clustered, as were the samples from the RBSL and RHSL varieties. As shown in Figure 5, the relative content of the most common upregulated lipids was higher in the soft-waxy varieties (RBSL and RHSL) than in the hard-crispy varieties. On the contrary, the relative content of three common downregulated lipids [MAG 16:1, MAG 18:2, and PG (14:0/20:2)] was lower in the soft-waxy varieties, which was consistent with the firmness characteristics of the fruit. The relative amount of these three downregulated lipids in the endocarp was in the order RBSL < RHSL < YBSL < YHSL. This indicated an essential role of these lipids in controlling the texture of guava fruit. Additionally, all parts of the fruit displayed steadily elevated levels of GPs, including PC (16:0/18:1), PC (18:1/18:1), and PE (18:1/18:2). Higher relative amounts of the three lipids were also detected in soft-waxy guavas compared with hard-crispy guavas. Of these, PCs and Pes were the most abundant in the GPs. GPs are vital in the cell membranes of fruits, PC is the major phospholipid, and PE significantly influences the fluidity/rigidity of membranes [36,37]. PG is crucial in the thylakoid membranes of chloroplasts, playing a vital role in photosynthesis, cell membrane structure, and mitochondrial function [38]. These phospholipids are essential for maintaining cell membrane fluidity and stability, which are fundamental for normal cell membrane structure and function. Alterations in lipid composition can impact the physical properties and biological traits of membranes, such as fruit firmness. Li et al. [21] demonstrated that higher PC levels improved phospholipid bilayer structure and cell membrane integrity in “Qiujin” apples, thus impacting fruit texture. Su et al. [22] observed decreasing phospholipid levels, including PCs and Pes, with the ripening and softening of bananas. Yu et al. [39] noted a significant impact of the composition and ratios of lipids in plant cell membranes on membrane fluidity, potentially influencing fruit firmness by increasing the rigidity of the lipid bilayer. These results were consistent with findings on guava. The significance of phospholipids, especially PCs and Pes, in shaping the lipid profile and distribution in guava fruits was highlighted, indicating that these lipids may influence the biological processes of the fruit.

### 3.5. Differential Lipid Enrichment Analysis

**Figure 6:**
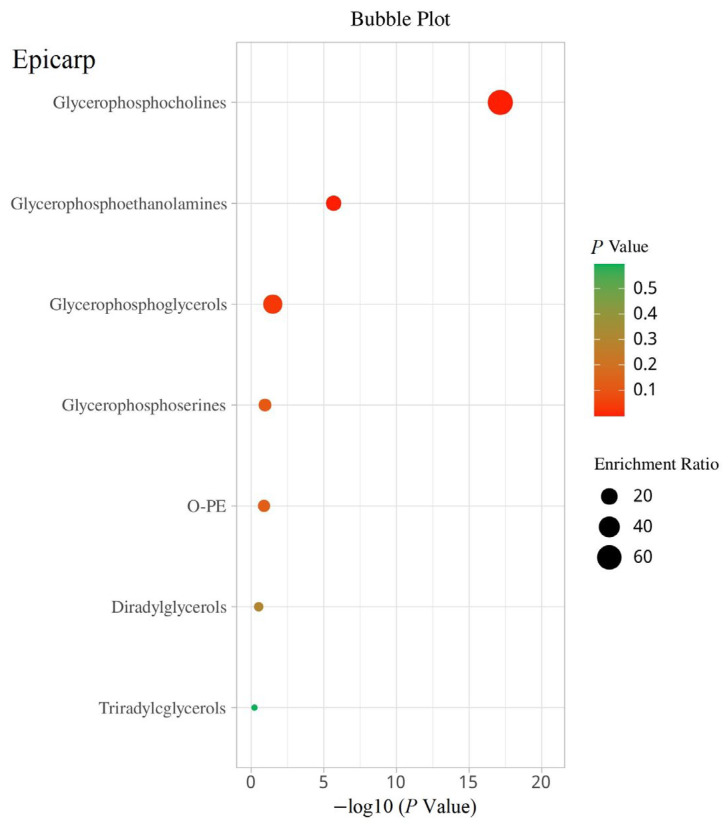

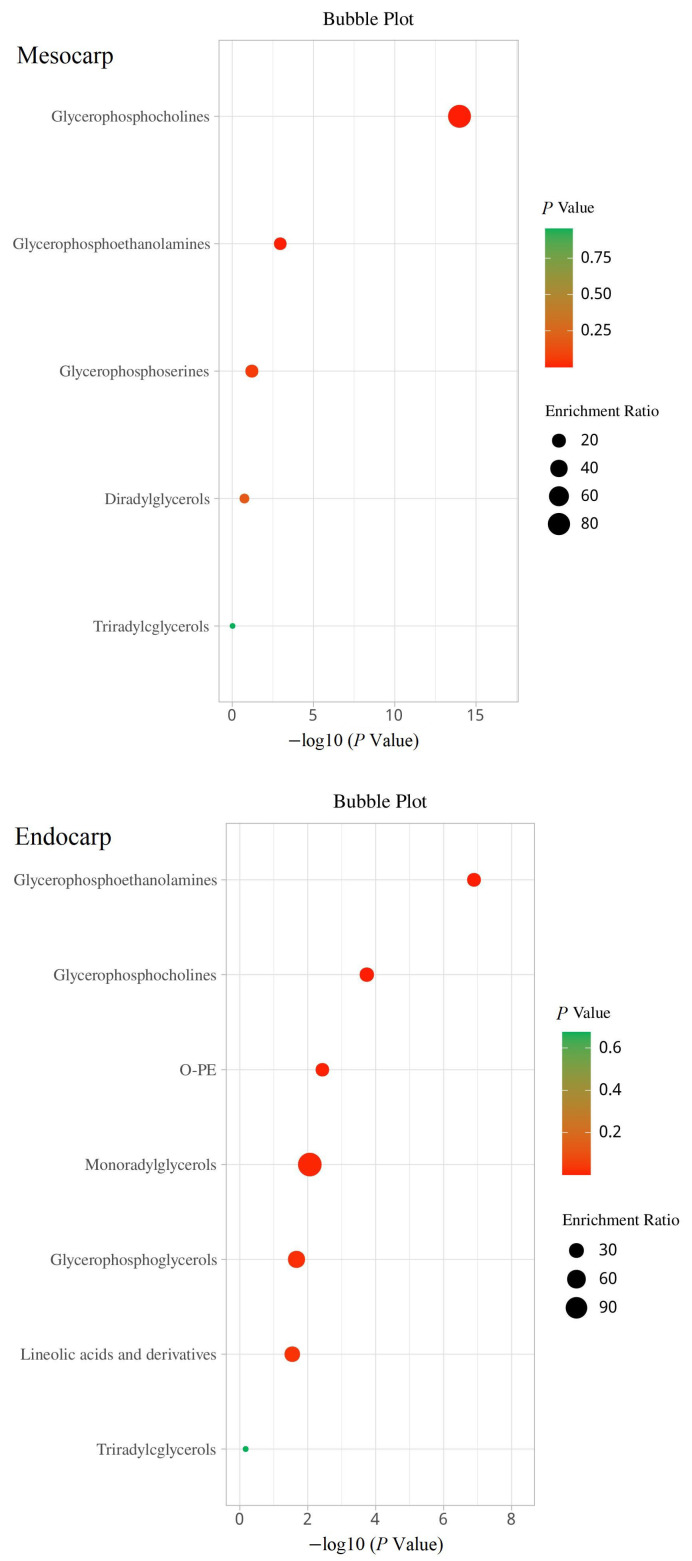
The bubble map of enrichment analysis of the common differential lipids by KEGG pathway in two comparison groups (YBSL vs. RBSL and YHSL vs. RHSL).

An enrichment analysis was conducted on the differentially common lipid metabolites for the two groups of guava fruits, namely, the hard-crispy and soft-waxy varieties (including white-fleshed and red-fleshed) (Figure 6, Tables S5 and S6). Of the 29 differential lipid metabolites detected in the epicarp, 26 were classified into seven metabolic pathways. Specifically, the glycerophosphocholine, glycerophosphoethanolamine, and glycerophosphoglycerol pathways displayed significant enrichment (*p* < 0.05), indicating their potential in epicarp formation. In the mesocarp, 15 of 21 differential lipid metabolites were annotated to five metabolic pathways, with significant enrichment in the glycerophosphocholine and glycerophosphoethanolamine pathways. In the endocarp, 17 of 18 differential lipid metabolites were annotated to seven metabolic pathways. However, no significant enrichment was observed in the triacylglycerol pathway. This result indicated that the metabolism of triacylglycerols was less active in the endocarp compared with other lipid metabolic pathways. In addition, the glycerophosphocholine and glycerophosphoethanolamine metabolic pathways were significantly enriched in all parts of the guava fruit. These pathways are known for their crucial role in modifying phospholipids during various physiological processes, influencing cell membrane dynamics and stress response mechanisms [24,33,40,41]. Moreover, the common differential lipids PC (16:0/18:1) and PC (18:1/18:1) were enriched in the glycerophosphocholine metabolic pathway, whereas the common differential lipid PE (18:1/18:2) was enriched in the glycerophosphoethanolamine metabolic pathway. Therefore, PC (16:0/18:1) and PC (18:1/18:1) may influence the morphological characteristics of guava formation universally. Future studies should investigate the regulatory mechanisms of these lipid metabolic pathways, particularly focusing on the common differential lipid PC (16:0/18:1), PC (18:1/18:1), and PE (18:1/18:2).

## 4. Conclusions

This study involved a multi-dimensional spatial analysis of lipid profiles in four guava varieties using UPLC-MS/MS. It provided a comprehensive analysis of the composition and abundance of 8242 lipid metabolites in the epicarp, mesocarp, and endocarp of the four guava varieties. The results indicated that GPs and GLs were the main lipid components of guava. The proportion of GPs was higher in soft-waxy guavas than in hard-crispy ones. Furthermore, compared with hard-crispy guavas, soft-waxy guavas had higher relative levels of upregulated lipids and lower levels of downregulated lipids in the endocarp. The lipids PC (16:0/18:1), PC (18:1/18:1), and PE (18:1/18:2) were upregulated across all guava parts, with higher relative abundance in soft-waxy guavas. These three lipids were significantly enriched in glycerophosphocholine and glycerophosphoethanolamine metabolic pathways. The results suggested that the lipids PC (16:0/18:1), PC (18:1/18:1), and PE (18:1/18:2) might influence fruit texture and serve as key biomarkers for distinguishing between hard-crispy and soft-waxy guavas. However, the biosynthetic pathways of these lipids and their functions in plant physiological processes need further exploration.

## Figures and Tables

**Table 1:** Morphological and physical characteristics of four varieties of guava fruit.

ID in Manuscript	Cultivar	Peel Color	Pulp Color	Fruit Texture	Horizontal Diameter (cm)	Vertical Diameter (cm)	Fruit Shape Index	Weight per Fruit (g)	Hardness (N)
YBSL	‘Zhenzhu’	Yellowish-green	White	Hard-crispy	6.40 ± 0.10 ^b^	6.49 ± 0.07 ^b^	1.01 ± 0.01 ^b^	152.89 ± 7.98 ^b^	5.82 ± 0.39 ^b^
RBSL	‘Bendi’	green	White	Soft-waxy	6.41 ± 0.04 ^b^	6.22 ± 0.05 ^c^	0.97 ± 0.01 ^c^	132.92 ± 8.14 ^c^	2.21 ± 0.55 ^c^
YHSL	‘Xiguahong’	Yellowish-green	Red	Hard-crispy	6.26 ± 0.06 ^c^	6.41 ± 0.05 ^b^	1.02 ± 0.01 ^b^	153.14 ± 7.98 ^b^	6.79 ± 0.22 ^a^
RHSL	‘Hongxin’	green	Red	Soft-waxy	7.28 ± 0.09 ^a^	8.10 ± 0.09 ^a^	1.11 ± 0.02 ^a^	179.56 ± 6.99 ^a^	2.51 ± 0.39 ^c^

Note: Data are presented as mean values ± SE (*n* = 10). Different letters in the same column indicate significant differences among the guava varieties (*p* < 0.05).

## Data Availability

The data presented in this study are available on request from the corresponding author.

## Supplementary Materials

The following supporting information can be downloaded at: https://www.mdpi.com/article/10.3390/foods14132330/s1, Table S1: Gradient elution conditions of mobile phase for the positive ion source; Table S2: Gradient elution conditions of mobile phase for the negative ion source; Table S3: Classification of lipids detected in guava; Table S4: Common differential lipids in the comparison groups of YBSL vs. RBSL and YHSL vs. RHSL; Table S5: Name/ID standardization for common differential lipids in the comparison groups of YBSL vs. RBSL and YHSL vs. RHSL; Table S6: Results of enrichment analysis for common differential lipids in the comparison groups of YBSL vs. RBSL and YHSL vs. RHSL.
