# Supplementary material for: Lipid Profiling of Four Guava Cultivars: A Multi-Dimensional Spatial Analysis
Source: Foods. 2025 Jun 30;14(13):2330. doi: 10.3390/foods14132330 (PMC12249162; doi:10.3390/foods14132330)
Supplement: Supplementary file 1 [file foods-14-02330-s001.zip › foods-3699206-supplementary.pdf]

Supplementary Material

Lipid Profiling of Four Guava Cultivars: A Multi-Dimensional Spatial Analysis

Qun Zhang [1, 2, 3], Xueren Cao [4], YuJun Ding [1,2], Chen Ma [1,2], Qiong Fan [1,3], Jia Song [1,5], Yu Rong [1, 3], Di Chen [1,3], Wenjiang Dong [6], Xiaopeng Wu [1], Zhi Xu[1, *] and Daizhu Lyu [1,2,3, *]

1 Analysis and Test Center, Chinese Academy of Tropical Agricultural Sciences, Haikou, Hainan 571101, China ; zhangqun123@zju.edu.cn (Q.Z.); dyjyj0822@163.com (Y.-J.D.); mc19860112@163.com (C.M.); joanhee@126.com (Q.F.); jia668837@163.com (J.S.); Amanda941130@163.com (Y.R.); chen-di_1008@yeah.net (D.C.); 373712474@qq.com (X.-P.W.)

2 Hainan Provincial Key Laboratory of Quality and Safety for Tropical Fruit and Vegetable Products, Haikou, Hainan 571101, China

3 Key Laboratory of Nutritional Quality and Health Benefits of Tropical Agricultural Products of Hai-kou City, Haikou 571101, China

4 Key Laboratory of Integrated Pest Management on Tropical Crops, Ministry of Agriculture, Environ-ment and Plant Protection Institute, Chinese Academy of Tropical Agricultural Sciences, Haikou 571101, China; caoxueren1984@163.com (X.-R.C.)

5 Key Laboratory of Quality and Safety Control for Subtropical Fruit and Vegetable,Ministry of Agri-culture and Rural Affairs, Haikou, Hainan 571101, China

6 Spice and Beverage Research Institute, Chinese Academy of Tropical Agricultural Sciences, Haikou, Hainan 571101, ChinaAffiliation; dongwenjiang.123@163.com (W.-J.D.)

Q.Z. and X.-R.C.contributed equally to this study.

*Correspondence: honic@yeah.net (Z.X.); ldz162000@126.com (D.-Z.L.)

Table S1. Gradient elution conditions of mobile phase for the positive ion source.

**Time （min）**	**Flow velocity （mL/min）**	**A (%)**	**B (%)**
0.00	0.3000	80.0	20.0
0.50	0.3000	80.0	20.0
1.50	0.3000	60.0	40.0
3.00	0.3000	40.0	60.0
13.00	0.3000	2.0	98.0
13.10	0.3000	80.0	20.0
17.00	0.3000	80.0	20.0

Table S2. Gradient elution conditions of mobile phase for the negative ion source.

**Time** (**min**)	**Flow velocity** (**mL/min**)	**A** (**%**)	**B** (**%**)
0.00	0.3000	0.0	100.0
2.00	0.3000	0.0	100.0
2.01	0.3000	0.0	100.0
11.00	0.3000	50.0	50.0
11.50	0.3000	70.0	30.0
12.50	0.3000	100.0	0.0
15.00	0.3000	100.0	0.0
15.01	0.3000	0.0	100.0
17.00	0.3000	0.0	100.0

Table S3. Classification of lipids detected in guava.

Lipids		Number of subclasses											
Class	Subclass	Epicarp				Mesocarp				Endocarp			
		YHSL	YBSL	RBSL	RHSL	YHSL	YBSL	RBSL	RHSL	YHSL	YBSL	RBSL	RHSL
GP	PE	132	125	124	125	123	129	117	114	119	122	118	116
	PS	67	59	63	68	67	68	60	67	68	65	62	67
	PC	65	67	65	69	66	68	71	65	66	69	71	71
	PG	59	56	59	61	55	58	63	55	59	60	57	55
	PA	58	54	56	62	61	59	54	58	57	54	60	60
	PI	51	52	53	48	48	51	52	46	49	51	52	53
	LPC	12	12	14	13	11	9	9	6	12	5	9	5
	LPE	10	13	13	10	6	9	10	9	6	9	10	9
	LPG	9	8	11	10	9	12	10	10	11	12	7	10
	LPS	9	9	10	10	7	8	7	6	6	8	9	6
	LPA	8	8	7	9	7	5	8	9	7	7	7	9
	LPI	7	9	8	7	7	6	8	7	7	8	8	8
GL	TAG	128	258	116	102	180	233	110	89	313	208	93	82
	DAG	35	24	7	14	42	18	8	17	42	14	12	23
	MAG	12	8	9	10	12	6	7	9	12	9	10	12
SP	HCER	8	4	5	5	8	6	5	7	8	3	6	8
	DCER	6	5	6	6	5	5	4	5	6	5	5	5
	SM	2	1	0	2	2	1	0	2	2	1	2	2
	CER	1	2	1	1	1	2	1	1	2	2	1	1
ST	CE	9	13	6	10	11	7	7	9	13	9	6	9

Note: YBSL, white-fleshed hard-crispy, ‘Zhenzhu’ guava; RBSL, white-fleshed soft-waxy guava, ‘Bendi’ guava; YHSL, red-fleshed hard-crispy guava, ‘Xiguahong’ guava; RHSL red-fleshed soft-waxy guava, ‘Hongxin’ guava; GP, glycerophospholipids; GL, glycerolipids; SP, sphingolipids; ST, sterols; PE, phosphatidylethanolamine; PS, phosphatidylserine; PC, phosphatidylcholine; PG, phosphatidylglycerol; PA, phosphatidic acid; PI, phosphatidylinositol; LPC, lysophosphatidylcholine; LPE, lysophosphatidylethanolamine; LPG, lysophosphatidylglycerol; LPS, lysophosphatidylserine; LPA, lysophosphatidic acid; LPI, lysophosphatidylinositol; TAG, triacylglycerol; DAG, diacylglycerol; MAG, monoacylglycerol; HCER, hexosylceramide; DCER, dihexosylceramide; CER, ceramide; SM, sphingomyelin; CE, cholesterol ester.

Table S4. Common differential lipids in the comparison groups of YBSL vs RBSL and YHSL vs RHSL.

Part	Number	Class	Lipid	YBSL vs RBSL log$_2$FC	YBSL vs RBSL Regulation type	YHSL vs RHSL log$_2$FC	YHSL vs RHSL Regulation type
Epicarp	1	GL	DAG(14:0/14:0)	-1.9084	down	4.5983	up
	2	GP	LPE(16:0)	-1.1341	down	1.2201	up
	3	GP	**PC(16:0/18:0)**	**1.2341**	**up**	**1.5998**	**up**
	4	GP	**PC(16:0/18:1)**	**1.699**	**up**	**2.4333**	**up**
	5	GP	PC(16:0/18:3)	-1.5948	down	4.3293	up
	6	GP	PC(16:0/20:1)	-1.5046	down	1.7233	up
	7	GP	**PC(16:1/18:2)**	**1.4887**	**up**	**1.9607**	**up**
	8	GP	**PC(18:0/18:1)**	**1.537**	**up**	**1.4664**	**up**
	9	GP	PC(18:0/18:3)	-1.2581	down	3.4414	up
	10	GP	**PC(18:1/16:1)**	**3.0069**	**up**	**1.0735**	**up**
	11	GP	**PC(18:1/18:1)**	**4.0451**	**up**	**1.3567**	**up**
	12	GP	**PC(18:1/18:2)**	**1.4578**	**up**	**2.6029**	**up**
	13	GP	PC(18:2/18:3)	-2.0012	down	3.9788	up
	14	GP	PE(16:0/18:3)	-1.92	down	2.4267	up
	15	GP	PE(16:0/20:1)	-1.0914	down	1.488	up
	16	GP	**PE(18:1/18:1)**	**2.205**	**up**	**1.9186**	**up**
	17	GP	**PE(18:1/18:2)**	**1.3318**	**up**	**2.0669**	**up**
	18	GP	PE(18:2/18:3)	-1.627	down	2.03	up
	19	GP	PE(O-16:0/18:2)	-1.3705	down	1.9375	up
	20	GP	PE(P-18:1/18:2)	-1.8795	down	1.5516	up

	21	GP	PG(16:0/18:3)	-2.9249	down	1.2914	up
	22	GP	PI(16:0/18:3)	-1.6468	down	4.5528	up
	23	GP	PI(18:2/16:1)	-1.3204	down	1.8906	up
	24	GP	PS(20:0/18:1)	-1.0279	down	1.3574	up
	25	GL	**TAG48:4**	**2.406**	**up**	**1.7086**	**up**
	26	GL	TAG53:3	1.7175	up	-1.7284	down
	27	GL	TAG53:4	1.7191	up	-1.7678	down
	28	GL	TAG55:4	2.6661	up	-1.8238	down
	29	GL	TAG55:5	2.6522	up	-1.8516	down
Mesocarp	1	GL	DAG(14:0/14:0)	-3.8526	down	3.3914	up
	2	GP	PC(14:0/18:2)	-2.9314	down	2.2284	up
	3	GP	**PC(16:0/18:0)**	**1.2333**	**up**	**1.8382**	**up**
	4	GP	**PC(16:0/18:1)**	**1.7392**	**up**	**2.3975**	**up**
	5	GP	PC(16:0/18:3)	-2.9772	down	2.2274	up
	6	GP	PC(16:0/20:1)	-2.6334	down	1.3561	up
	7	GP	PC(18:0/18:3)	-2.5273	down	1.5798	up
	8	GP	**PC(18:1/18:1)**	**4.4597**	**up**	**1.533**	**up**
	9	GP	PC(18:2/18:3)	-3.7255	down	1.6047	up
	10	GP	PE(16:0/20:2)	-1.7412	down	1.4784	up
	11	GP	**PE(18:1/18:2)**	**1.2969**	**up**	**1.1855**	**up**
	12	GP	PE(18:2/20:2)	-1.8246	down	1.2473	up
	13	GP	PG(14:0/18:2)	-1.4504	down	1.0759	up
	14	GP	PG(18:0/14:0)	-1.1825	down	1.254	up
	15	GP	PI(14:0/18:3)	-2.0193	down	1.0682	up
	16	GP	PI(16:0/18:3)	-3.0074	down	2.3431	up

	17	GP	PI(18:2/16:1)	-2.7834	down	1.4781	up
	18	GP	**PI(20:0/18:1)**	**1.3597**	**up**	**1.5329**	**up**
	19	GP	PI(20:0/18:3)	-1.709	down	1.3634	up
	20	GP	PS(18:1/18:2)	-1.204	down	1.4941	up
	21	GL	**TAG58:9**	**1.6175**	**up**	**1.5295**	**up**
Endocarp	1	GP	**LPE(16:0)**	**1.8114**	**up**	**4.4078**	**up**
	2	GP	**LPE(18:2)**	**1.27**	**up**	**3.6395**	**up**
	3	GL	**MAG 16:1**	**-2.0779**	**down**	**-2.0345**	**down**
	4	GL	**MAG 18:2**	**-1.7567**	**down**	**-3.4422**	**down**
	5	GP	**PC(16:0/18:1)**	**2.6414**	**up**	**2.4199**	**up**
	6	GP	**PC(18:1/18:1)**	**1.7007**	**up**	**2.8201**	**up**
	7	GP	**PC(20:0/18:1)**	**1.2595**	**up**	**1.8829**	**up**
	8	GP	**PE(18:0/16:0)**	**1.7372**	**up**	**1.1462**	**up**
	9	GP	**PE(18:0/18:3)**	**1.4342**	**up**	**1.8803**	**up**
	10	GP	**PE(18:1/18:1)**	**1.6929**	**up**	**2.1774**	**up**
	11	GP	**PE(18:1/18:2)**	**2.3952**	**up**	**2.388**	**up**
	12	GP	**PE(O-18:0/18:1)**	**1.929**	**up**	**1.6909**	**up**
	13	GP	**PE(O-18:0/18:2)**	**2.4136**	**up**	**2.2971**	**up**
	14	GP	**PG(14:0/20:2)**	**-1.6684**	**down**	**-2.5903**	**down**
	15	GP	**PG(18:2/16:1)**	**1.4382**	**up**	**1.5366**	**up**
	16	GL	**TAG46:2**	**1.5432**	**up**	**2.067**	**up**
	17	GL	**TAG52:8**	**1.5683**	**up**	**2.1923**	**up**
	18	GL	TAG56:10	2.3964	up	-2.0337	down

Note: The lipids with names highlighted in red font are commonly upregulated, those in purple font are commonly upregulated and present in all guava parts, and those

in blue font are commonly downregulated.

Table S5. Name/ID standardization for common differential lipids in the comparison groups of YBSL vs RBSL and YHSL vs RHSL.

Part	Number	Query	Hit	HMDB	PubChem	KEGG
Epicarp	1	DAG(14:0/14:0)	DG(14:0/14:0/0:0)	HMDB0007008	10369168	C16667
	2	LPE(16:0)	LysoPE(0:0/16:0)	HMDB0011473	53480922	-
	3	PC(16:0/18:0)	PC(16:0/18:0)	HMDB0007970	24778686	C00157
	4	PC(16:0/18:1)	PC(16:0/18:1(11Z))	HMDB0007971	24778688	C00157
	5	PC(16:0/18:3)	PC(16:0/18:3(6Z,9Z,12Z))	HMDB0007974	24778699	C00157
	6	PC(16:0/20:1)	PC(16:0/20:1(11Z))	HMDB0007978	52922418	C00157
	7	PC(16:1/18:2)	PC(16:1(9Z)/18:2(9Z,12Z))	HMDB0008006	24778768	C00157
	8	PC(18:0/18:1)	PC(18:0/18:1(11Z))	HMDB0008037	24778815	C00157
	9	PC(18:0/18:3)	PC(18:0/18:3(6Z,9Z,12Z))	HMDB0008040	52922655	C00157
	10	PC(18:1/16:1)	PC(18:1(11Z)/16:1(9Z))	HMDB0008068	53478719	C00157
	11	PC(18:1/18:1)	PC(36:2)	HMDB0000593	10350317	C00157
	12	PC(18:1/18:2)	PC(18:1(11Z)/18:2(9Z,12Z))	HMDB0008072	53478723	C00157
	13	PC(18:2/18:3)	PC(18:2(9Z,12Z)/18:3(6Z,9Z,12Z))	HMDB0008140	52922729	C00157
	14	PE(16:0/18:3)	PE(16:0/18:3(6Z,9Z,12Z))	HMDB0008929	52924922	C00350
	15	PE(16:0/20:1)	PE(16:0/20:1(11Z))	HMDB0008933	9547010	C00350
	16	PE(18:1/18:1)	PE(18:1(11Z)/18:1(11Z))	HMDB0009025	53479624	C00350
	17	PE(18:1/18:2)	PE(18:1(11Z)/18:2(9Z,12Z))	HMDB0009027	53479626	C00350
	18	PE(18:2/18:3)	PE(18:2(9Z,12Z)/18:3(6Z,9Z,12Z))	HMDB0009094	52924365	C00350
	19	PE(O-16:0/18:2)	PE(O-16:0/18:2)	-	-	-
	20	PE(P-18:1/18:2)		-	-	-
	21	PG(16:0/18:3)	PG(16:0/18:3(6Z,9Z,12Z))	HMDB0010576	52927171	-
	22	PI(16:0/18:3)	PI(16:0/18:3(6Z,9Z,12Z))	-	52928330	-

	23	PI(18:2/16:1)	PI(18:2(9Z,12Z)/16:1(9Z))	-	52927767	-
	24	PS(20:0/18:1)	PS(20:0/18:1(11Z))	HMDB0112517	131819799	-
	25	TAG48:4	TG(14:0/14:0/20:4(5Z,8Z,11Z,14Z))	HMDB0042080	131753226	-
	26	TAG53:3	TG(15:0/18:0/20:3(5Z,8Z,11Z))	HMDB0043058	131754189	-
	27	TAG53:4	TG(15:0/16:0/22:4(7Z,10Z,13Z,16Z))	HMDB0043038	131754169	-
	28	TAG55:4	TG(15:0/18:0/22:4(7Z,10Z,13Z,16Z))	HMDB0043067	131754197	-
	29	TAG55:5	TG(15:0/18:0/22:5(4Z,7Z,10Z,13Z,16Z))	HMDB0043068	131754198	
Mesocarp	1	DAG(14:0/14:0)	DG(14:0/14:0/0:0)	HMDB0007008	10369168	C16667
	2	PC(14:0/18:2)	PC(32:2)	HMDB0007874	24778624	C00157
	3	PC(16:0/18:0)	PC(16:0/18:0)	HMDB0007970	24778686	C00157
	4	PC(16:0/18:1)	PC(16:0/18:1(11Z))	HMDB0007971	24778688	C00157
	5	PC(16:0/18:3)	PC(16:0/18:3(6Z,9Z,12Z))	HMDB0007974	24778699	C00157
	6	PC(16:0/20:1)	PC(16:0/20:1(11Z))	HMDB0007978	52922418	C00157
	7	PC(18:0/18:3)	PC(18:0/18:3(6Z,9Z,12Z))	HMDB0008040	52922655	C00157
	8	PC(18:1/18:1)	PC(36:2)	HMDB0000593	10350317	C00157
	9	PC(18:2/18:3)	PC(18:2(9Z,12Z)/18:3(6Z,9Z,12Z))	HMDB0008140	52922729	C00157
	10	PE(16:0/20:2)	PE(16:0/20:2(11Z,14Z))	HMDB0008934	52924213	C00350
	11	PE(18:1/18:2)	PE(18:1(11Z)/18:2(9Z,12Z))	HMDB0009027	53479626	C00350
	12	PE(18:2/20:2)	PE(18:2(9Z,12Z)/20:2(11Z,14Z))	HMDB0009099	52924372	C00350
	13	PG(14:0/18:2)	PG(14:0/18:2(9Z,12Z))	-	52927192	-
	14	PG(18:0/14:0)	PG(18:0/14:0)	-	9547121	-
	15	PI(14:0/18:3)	PI(14:0/18:3(6Z,9Z,12Z))	-	52927520	-
	16	PI(16:0/18:3)	PI(16:0/18:3(6Z,9Z,12Z))	-	52928330	-
	17	PI(18:2/16:1)	PI(18:2(9Z,12Z)/16:1(9Z))	-	52927767	-
	18	PI(20:0/18:1)	PI(20:0/18:1(9Z))	-	52928376	-

	19	PI(20:0/18:3)	PI(20:0/18:3(6Z,9Z,12Z))	-	52927939	-
	20	PS(18:1/18:2)	PS(18:1(9Z)/18:2(9Z,12Z))	HMDB0012391	52926065	-
	21	TAG58:9	TG(18:1(9Z)/20:4(5Z,8Z,11Z,14Z)/20:4(5Z,8Z,11Z,14Z))	HMDB0005463	9545200	C00422
Endocarp	1	LPE(16:0)	LysoPE(0:0/16:0)	HMDB0011473	53480922	-
	2	LPE(18:2)	LysoPE(0:0/18:2(9Z,12Z))	HMDB0011477	53480926	-
	3	MAG 16:1	MG(0:0/16:1(9Z)/0:0)	HMDB0011534	53480960	-
	4	MAG 18:2	MG(0:0/18:2(9Z,12Z)/0:0)	HMDB0011538	5365676	-
	5	PC(16:0/18:1)	PC(16:0/18:1(11Z))	HMDB0007971	24778688	C00157
	6	PC(18:1/18:1)	PC(36:2)	HMDB0000593	10350317	C00157
	7	PC(20:0/18:1)	PC(20:0/18:1(11Z))	HMDB0008268	24779033	C00157
	8	PE(18:0/16:0)	PE(18:0/16:0)	HMDB0008989	445757	C00350
	9	PE(18:0/18:3)	PE(18:0/18:3(6Z,9Z,12Z))	HMDB0008995	52924329	C00350
	10	PE(18:1/18:1)	PE(18:1(11Z)/18:1(11Z))	HMDB0009025	53479624	C00350
	11	PE(18:1/18:2)	PE(18:1(11Z)/18:2(9Z,12Z))	HMDB0009027	53479626	C00350
	12	PE(O-18:0/18:1)	PE(O-18:0/18:1)	-	-	-
	13	PE(O-18:0/18:2)	PE(O-18:0/18:2)	-	-	-
	14	PG(14:0/20:2)	PG(14:0/20:2(11Z,14Z))	-	52926368	-
	15	PG(18:2/16:1)	PG(18:2(9Z,12Z)/16:1(9Z))	HMDB0010646	52926611	-
	16	TAG46:2	TG(16:1(9Z)/14:0/16:1(9Z))	HMDB0010419	53480484	-
	17	TAG52:8	TG(14:0/18:3(6Z,9Z,12Z)/20:5(5Z,8Z,11Z,14Z,17Z))	HMDB0042567	131753704	-
	18	TAG56:10	TG(18:3(9Z,12Z,15Z)/18:2(9Z,12Z)/20:5(5Z,8Z,11Z,14Z,17Z))	HMDB0010508	53480555	

Note: "-" is for no match.

Table S6. Results of enrichment analysis for common differential lipids in the comparison groups of YBSL vs RBSL and YHSL vs RHSL.

Parts	Metabolite Set	Total	Hits	P value	Metabolites
Epicarp	Glycerophosphocholines	1350	11	7E-18	PC(36:2); PC(16:0/18:0); PC(16:0/18:1(11Z)); PC(16:0/18:3(6Z,9Z,12Z)); PC(16:0/20:1(11Z)); PC(16:1(9Z)/18:2(9Z,12Z)); PC(18:0/18:1(11Z)); PC(18:0/18:3(6Z,9Z,12Z)); PC(18:1(11Z)/16:1(9Z)); PC(18:1(11Z)/18:2(9Z,12Z)); PC(18:2(9Z,12Z)/18:3(6Z,9Z,12Z))
	Glycerophosphoethanolamines	3080	6	2E-06	PE(16:0/18:3(6Z,9Z,12Z)); PE(16:0/20:1(11Z)); PE(18:1(11Z)/18:1(11Z)); PE(18:1(11Z)/18:2(9Z,12Z)); PE(18:2(9Z,12Z)/18:3(6Z,9Z,12Z)); LysoPE(0:0/16:0)
	Triradylcglycerols	39900	5	0.596	TG(14:0/14:0/20:4(5Z,8Z,11Z,14Z)); TG(15:0/16:0/22:4(7Z,10Z,13Z,16Z)); TG(15:0/18:0/20:3(5Z,8Z,11Z)); TG(15:0/18:0/22:4(7Z,10Z,13Z,16Z)); TG(15:0/18:0/22:5(4Z,7Z,10Z,13Z,16Z))
	Diradylglycerols	2830	1	0.305	DG(14:0/14:0/0:0)
	Glycerophosphoglycerols	260	1	0.0327	PG(16:0/18:3(6Z,9Z,12Z))
	Glycerophosphoserines	929	1	0.112	PS(20:0/18:1(11Z))
	O-PE	1100	1	0.131	PE(O-16:0/18:2)
Mesocarp	Glycerophosphocholines	1350	8	1E-14	PC(36:2); PC(32:2); PC(16:0/18:0); PC(16:0/18:1(11Z)); PC(16:0/18:3(6Z,9Z,12Z)); PC(16:0/20:1(11Z)); PC(18:0/18:3(6Z,9Z,12Z)); PC(18:2(9Z,12Z)/18:3(6Z,9Z,12Z))
	Glycerophosphoethanolamines	3080	3	0.0011	PE(16:0/20:2(11Z,14Z)); PE(18:1(11Z)/18:2(9Z,12Z)); PE(18:2(9Z,12Z)/20:2(11Z,14Z))
	Diradylglycerols	2830	1	0.178	DG(14:0/14:0/0:0)
	Glycerophosphoserines	929	1	0.062	PS(18:1(9Z)/18:2(9Z,12Z))
	Triradylcglycerols	39900	1	0.953	TG(18:1(9Z)/20:4(5Z,8Z,11Z,14Z)/20:4(5Z,8Z,11Z,14Z))
Endocarp	Glycerophosphoethanolamines	3080	6	1E-07	PE(18:0/16:0); PE(18:0/18:3(6Z,9Z,12Z)); PE(18:1(11Z)/18:1(11Z)); PE(18:1(11Z)/18:2(9Z,12Z)); LysoPE(0:0/16:0); LysoPE(0:0/18:2(9Z,12Z))
	Glycerophosphocholines	1350	3	0.0002	PC(36:2); PC(16:0/18:1(11Z)); PC(20:0/18:1(11Z))

Triradylcglycerols	39900	3	0.675	TG(16:1(9Z)/14:0/16:1(9Z)); TG(18:3(9Z,12Z,15Z)/18:2(9Z,12Z)/20:5(5Z,8Z,11Z,14Z,17Z)); TG(14:0/18:3(6Z,9Z,12Z)/20:5(5Z,8Z,11Z,14Z,17Z))
O-PE	1100	2	0.0037	PE(O-18:0/18:1); PE(O-18:0/18:2)
Glycerophosphoglycerols	260	1	0.0215	PG(18:2(9Z,12Z)/16:1(9Z))
Lineolic acids and derivatives	348	1	0.0286	MG(0:0/18:2(9Z,12Z)/0:0)
Monoradylglycerols	105	1	0.0087	MG(0:0/16:1(9Z)/0:0)
